# Fetal Growth Restriction and Subsequent Low Grade Fetal Inflammatory Response Are Associated with Early-Onset Neonatal Sepsis in the Context of Early Preterm Sterile Intrauterine Environment

**DOI:** 10.3390/jcm10092018

**Published:** 2021-05-08

**Authors:** Kyung Chul Moon, Chan-Wook Park, Joong Shin Park, Jong Kwan Jun

**Affiliations:** 1 Department of Pathology, Seoul National University College of Medicine, Seoul 03080, Korea; blue7270@gmail.com; 2 Department of Obstetrics and Gynecology, Seoul National University College of Medicine, Seoul 03080, Korea; jsparkmd@snu.ac.kr (J.S.P.); jhs0927@snu.ac.kr (J.K.J.); 3 Institute of Reproductive Medicine and Population, Seoul National University Medical Research Center, Seoul 03080, Korea

**Keywords:** early-onset neonatal sepsis, fetal growth restriction, low-grade fetal inflammatory response, sterile intrauterine environment, preterm birth

## Abstract

There is no information about whether fetal growth restriction (FGR) is an independent risk factor for low-grade fetal inflammatory response (FIR), and which is more valuable for the prediction of early-onset neonatal sepsis (EONS) between low-grade FIR or fetal inflammatory response syndrome (FIRS) in the context of human early preterm sterile intrauterine environment. We examined FIR (umbilical cord plasma (UCP) CRP concentration at birth) according to the presence or absence of FGR (birth weight < 5th percentile for gestational age (GA)) and EONS in 81 singleton preterm births (GA at delivery: 24.5~33.5 weeks) within 72 h after amniocentesis and with sterile intrauterine environment. A sterile intrauterine environment was defined by the presence of both a sterile amniotic fluid (AF) (AF with both negative culture and MMP-8 < 23 ng/mL) and inflammation-free placenta. Median UCP CRP (ng/mL) was higher in cases with FGR than in those without FGR (63.2 vs. 34.5; *p* = 0.018), and FGR was an independent risk factor for low-grade FIR (UCP CRP ≥ 52.8 ng/mL) (OR 3.003, 95% CI 1.024–8.812, *p* = 0.045) after correction for confounders. Notably, low-grade FIR (positive likelihood-ratio (LR) and 95% CI, 2.3969 (1.4141–4.0625); negative-LR and 95% CI, 0.4802 (0.2591–0.8902)), but not FIRS (positive-LR and 95% CI, 2.1071 (0.7526–5.8993); negative-LR and 95% CI, 0.8510 (0.6497–1.1145)), was useful for the identification of EONS. In conclusion, FGR is an independent risk factor for low-grade FIR, and low-grade FIR, but not FIRS, has a value for the identification of EONS in the context of the early preterm sterile intrauterine environment.

## 1. Introduction

FGR frequently develops due to utero-placental insufficiency [1,2], which is associated with chronic hypoxia at the feto–maternal interface [3,4,5]. Therefore, non-infection-related “danger-signals” such as chronic hypoxia in FGR are likely to trigger a non-specific low-grade inflammation as a host-defense mechanism in the fetus. Indeed, recent animal studies reported that chronic hypoxemia-absent bacterial infection resulted in mildly growth-restricted offspring [6,7] and increased IL-6 and TNF-α proteins and mRNAs to mild to moderate levels in fetal sera [6,8] and organs (i.e., lung, heart and brain) [6,7,9,10,11,12,13] (Table 1). However, the relationship between FGR and fetal inflammation is still controversial in a substantial amount of human studies [14,15,16,17,18,19] (Table 2). FGR was associated with an elevated fetal inflammatory response (FIR) in some human studies [16,18,19], but other human studies did not display this relationship [14,15,17]. Moreover, it should be noted that all of these human studies have a critical limitation, which is that they did not exclude neonates with ascending intrauterine infection (AIUI) (i.e., intraamniotic infection/inflammation or acute histologic chorioamnionitis [acute-HCA]/funisitis), thereby failing to remove critical sources of bias resulting in the development of FIR in cases with unsearched infection/inflammation in amniotic fluid (AF) and placenta [14,15,16,17,18,19] (Table 2). Therefore, these previous human studies were unlikely to be adequate to explore the pure effect of FGR on the development of FIR [14,15,16,17,18,19] (Table 2), while chronic hypoxemia-absent bacterial infection and subsequent development of FGR could be associated with an increased FIR in controlled animal experiments [6,7,8,9,10,11,12,13] (Table 1). Fetal inflammatory response syndrome (FIRS) defined as an elevation of either fetal plasma IL-6 or C-reactive protein (CRP) above a specific cutoff value (i.e., IL-6 ≥ 11 pg/mL, CRP ≥ 200 ng/mL) [20,21] is thought as the fetal counterpart of systemic inflammatory response syndrome (SIRS) in adults [22,23]. We used CRP as a marker for FIR at the year of 2003 based on the following reasons [24,25,26,27,28,29,30,31]: (1) assay of CRP has the advantage over cytokine determination (i.e., IL-6) in that it is widely available in most clinical laboratories; (2) the sensitivity of umbilical cord plasma (UCP) CRP in the identification of positive AF culture, early-onset neonatal sepsis (EONS) and funisitis was similar to that of IL-6, and moreover, the specificity of CRP in the identification of EONS and funisitis was significantly higher than that of IL-6 [21]; and (3) there was a strong correlation between UCP CRP and IL-6 concentrations [21]. FIRS has been unexceptionally reported in fetuses associated with either intraamniotic infection/inflammation or acute-HCA/funisitis [20,21,22,23,25,32], while SIRS can occur either in the setting of sepsis due to infection or in a wide variety of circumstances where sterile processes (i.e., ischemia, trauma or several insults) activate inflammation [33,34,35]. Unfortunately, there is no human information about the development of either low-grade FIR or FIRS in preterm gestation not associated with AIUI. However, given that chronic hypoxemia-absent bacterial infection results in mildly growth restricted offspring and mild to moderate FIR in animal studies [6,7] and FIRS is the fetal counterpart of SIRS in adults, there is a good chance that FGR in the sterile intrauterine environment of human preterm gestation is associated with either low-grade FIR or FIRS.

It is not yet known which mechanism is involved in the development of early-onset neonatal sepsis (EONS) in newborns with the history of FGR, although FGR has been reported to be associated with EONS. However, it is plausible that either low-grade FIR or FIRS can be an important pathophysiologic mechanism for the subsequent development of EONS in newborns with FGR if it is proven that FGR in the sterile intrauterine environment of human preterm gestation is a risk factor for either low-grade FIR or FIRS. We did not find any human reports about whether FGR is a risk factor for either low-grade FIR or FIRS, and either low-grade FIR or FIRS is associated with EONS in preterm gestation not associated with AIUI. We hypothesized that FGR is an independent risk factor for low-grade FIR, and low-grade FIR, but not FIRS, has a value for the identification of EONS in the context of early preterm sterile intrauterine environment. This study was performed to test these issues.

## 2. Materials and Methods

### 2.1. Study Design and Patient Population

**Figure 1:**
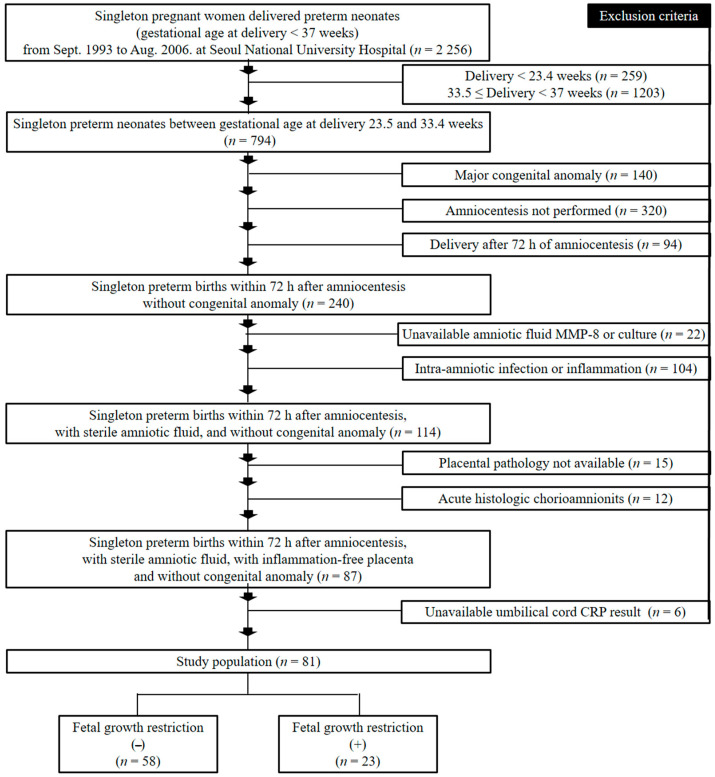
Flow chart of the study population.

Umbilical cord plasma (UCP) CRP levels at birth were examined in 81 patients who delivered singleton-neonates at Seoul National University Hospital between September 1993 and August 2006 and who met the following criteria: (1) GA at delivery between 24.5 and 33.4 weeks; (2) no major congenital anomalies; (3) amniocentesis-to-delivery interval <72 h; (4) sterile AF; (5) inflammation-free placenta; and (6) available UCP CRP results at birth (Figure 1).

An amniocentesis-to-delivery interval of less than 72 h was used as a criterion for the meaningful temporal relationship between the presence of sterile AF and UCP CRP concentration at birth. Sterile AF was defined as AF without both infection and inflammation. UCP CRP levels at delivery were compared between cases with and without FGR, and between those with and without EONS. Trans-abdominal amniocentesis was routinely offered to identify AF infection and inflammation to all pregnant women who were admitted in our hospital with the diagnosis of preterm labor and intact membranes (PTL) and preterm premature rupture of membranes (preterm-PROM), and was also performed to assess fetal lung maturity in patients with maternal fetal indication such as preeclampsia and idiopathic FGR. Moreover, placenta and umbilical cord were examined to assess the acute inflammatory changes in all pregnant women who delivered preterm neonates. PTL was defined as the presence of regular uterine contractions with a frequency of at least two every 10 min and cervical changes in the context of intact membranes, and rupture of membranes (ROM) was diagnosed by examination with sterile speculum confirming pooling of AF in the vagina, a positive nitrazine paper test results and a positive ferning test result as previously reported [36,37]. The Institutional Review Board of Seoul National University Hospital approved the collection and use of these samples and information for research purposes, and written informed consent was obtained from all study subjects. The Institutional Review Board of Seoul National University Hospital specifically approved this study (12 December 2016; IRB no. 1612-023-812).

### 2.2. Clinical Characteristics, Fetal Growth Restriction (FGR) and Early Onset Neonatal Sepsis (EONS)

We investigated maternal age, parity, GA at amniocentesis, antenatal use of corticosteroids and antibiotics, route of delivery, gender of newborn, causes of preterm delivery, GA at delivery, birth weight, 1 min and 5 min Apgar score, and umbilical arterial pH at birth as demographic and clinical characteristics. GA was defined according to the criteria suggested by American College of Obstetricians and Gynecologists [38]. Based on substantial evidence about the relationship between perinatal morbidity/mortality and birth weight (BW) < the 5th percentile at each GA [39,40], FGR was diagnosed when BW was less than the 5th percentile at each GA according to the previously published criteria [41]. EONS was proved in the presence of a positive blood culture result within 72 h of delivery [25], and was suspected in the absence of a positive culture when clinical suspicion of sepsis was present (i.e., lethargy, apnea, respiratory distress, hypoperfusion or shock), antibiotics treatment was performed for ≥5 days [42], and two or more were obtained among the following items: (1) WBC < 5000 cells/mm^3^; (2) polymorpholeukocytes <1800 cells/mm^3^; and (3) bands neutrophils/total neutrophils >0.2 [28]. These diagnostic criteria were previously used in the literature [28,42]. EONS was defined as the presence of proven or suspected EONS in the current study.

### 2.3. Placental Pathologic Examination

Placental tissue samples included all placental compartments (i.e., extra-placental membranes (chorio-decidua and amnion), chorionic plate and umbilical cord), and were processed for pathologic evaluation. Acute-HCA (i.e., chorio-deciduitis, amnionitis, chorionic plate inflammation) and funisitis were diagnosed according to previously published criteria [43] as follows; (1) chorio-deciduitis was diagnosed in the presence of at least 1 focus of >5 neutrophils in chorio-decidua; (2) amnionitis was diagnosed in the presence of at least 1 focus of >5 neutrophils in amnion; (3) chorionic plate inflammation was diagnosed in the presence of >1 focus of at least 10 neutrophilic collections or diffuse inflammation in subchorionic fibrin, and/or diffuse and dense inflammation, neutrophilic infiltration into connective tissue of placental plate, or placental vasculitis, and (4) funisitis was diagnosed in the presence of neutrophil infiltration into the umbilical vessel walls or Wharton’s jelly. Inflammatory-free placenta was defined when there was no inflammatory change in any placental compartments (i.e., chorio-decidua, amnion, chorionic plate and umbilical cord). Pathologists were blinded to clinical information.

### 2.4. Amniotic Fluid (AF) Infection and Inflammation

AF was cultured for aerobic and anaerobic bacteria and genital mycoplasma (Ureaplasma urelyticum and Mycoplasma hominis) according to the previous published method [37,44]. The remaining AF was processed, and matrix metalloproteinase-8 (MMP-8) levels in stored AF were examined with a commercially available enzyme-linked immunosorbent assay (Amersham Pharmacia Biotech Inc., Little Chalfont, Bucks, UK) according to the methods previously described [44,45]. The sensitivity of the test was <0.3 ng/mL. Both intra- and inter-assay coefficients of variation were <10%. Details about this MMP-8 assay and its performance were also concretely provided in previous reports [44,45]. AF inflammation was diagnosed when an increased MMP-8 concentration of more than 23 ng/mL was present in AF according to the previous published criteria [44,46].

### 2.5. Fetal Inflammatory Response (FIR), Low-Grade FIR and Fetal Inflammatory Response Syndrome (FIRS)

FIR was determined by UCP CRP concentration at birth. Umbilical cord blood was collected and processed, and CRP concentrations in stored cord plasma were measured according to the methods previously described [21]. The CRP assay and its performance were also concretely provided in a previous report [21]. Low-grade FIR was newly diagnosed as an increased UCP CRP concentration at birth more than the cut-off value (≥52.8 ng/mL) selected with the use of a receiver operating characteristic (ROC) curve for the identification of EONS in the context of early preterm sterile intrauterine milieu. Our group previously suggested a cut-off value (200 ng/mL) of UCP CRP concentration at birth for the identification of funisitis (regarded as the histologic counterpart of FIRS) in preterm study population [21], and therefore, we defined FIRS as an increased UCP CRP concentration at birth (≥200 ng/mL) according to our previous report [21].

### 2.6. Statistical Analysis

We used the Mann–Whitney U test for the comparison of continuous variables, and Fisher’s exact test to compare the proportions in clinical and pregnant information of study population. The Mann–Whitney U test and Fisher’s exact test were used for the comparison of UCP CRP levels at birth and the frequencies of low-grade FIR and FIRS, respectively, between the presence or absence of FGR and between the presence or absence of EONS. We used Pearson’s chi-square test and the linear by linear association test to compare the frequency of EONS between groups according to the presence or absence of FGR and/or low-grade FIR (i.e., FGR[−]/low-grade FIR[−], FGR[+]/low-grade FIR[−], FGR[−]/low-grade FIR[+], and FGR[+]/low-grade FIR[+]) and according to the presence or absence of FGR and/or FIRS (i.e., FGR[−]/FIRS[−], FGR[+]/FIRS[−], FGR[−]/FIRS[+], and FGR[+]/FIRS[+]). Logistic regression analysis was performed to evaluate the contributing factors to the occurrence of low-grade FIR and FIRS. We calculated the diagnostic indices, predicted values (PVs) and likelihood ratios (LRs) of low-grade FIR and FIRS for the identification of EONS. *p* < 0.05 was thought to be significant.

## 3. Results

### 3.1. Clinical and Pregnant Information According to the Presence or Absence of Fetal Growth Restriction (FGR) and Early Onset Neonatal Sepsis (EONS)

FGR was present in 28.4% (23/81) of patients and EONS was found in 25.3% (20/79) of patients. Table 3 and Table 4 demonstrate clinical and pregnant information according to the presence or absence of FGR and EONS, respectively. Neonates with EONS had a significantly lower GA at delivery and higher frequency of FGR than those without EONS (Table 4).

### 3.2. Umbilical Cord Plasma (UCP) CRP Concentrations at Birth According to the Presence or Absence of Fetal Growth Restriction (FGR) and Early Onset Neonatal Sepsis (EONS)

**Figure 2:**
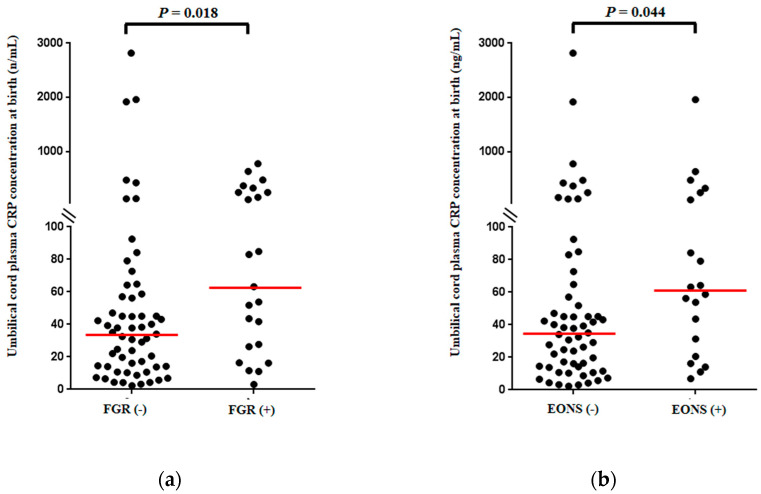
Umbilical cord plasma (UCP) CRP concentrations at birth according to the presence or absence of fetal growth restriction (FGR) (median, (interquartile-range (IQR)); 63.2 (220.60) vs. 34.5 (43.47) ng/mL; *p* = 0.018) (**a**) and early-onset neonatal sepsis (EONS) (median, (IQR); 60.95 (190.08) vs. 35.0 (50.60) ng/mL; *p* = 0.044) (**b**). Of 81 cases which met the entry for this study, two neonates were excluded from the analysis in the evaluation of EONS because they died shortly after delivery as a result of extreme prematurity and thus only 79 neonates could be evaluated with respect to the presence or absence of EONS.

Figure 2 shows UCP CRP concentrations at birth in neonates with or without FGR (Figure 2a) and EONS (Figure 2b). Preterm-neonates with FGR had a significantly higher median UCP CRP concentration (ng/mL) at birth than those without FGR (Figure 2a) (median, (interquartile-range (IQR)); 63.2 (220.60) vs. 34.5 (43.47); *p* = 0.018). Moreover, UCP CRP concentrations (ng/mL) at birth were significantly higher in preterm neonates with EONS than in those without EONS (Figure 2b) (median, (IQR); 60.95 (109.08) vs. 35.0 (50.60) ng/mL; *p* = 0.044).

### 3.3. Low-Grade Fetal Inflammatory Response (FIR) and Fetal Inflammatory Response Syndrome (FIRS) According to the Presence or Absence of Fetal Growth Restriction (FGR) and Early-Onset Neonatal Sepsis (EONS)

**Figure 3:**
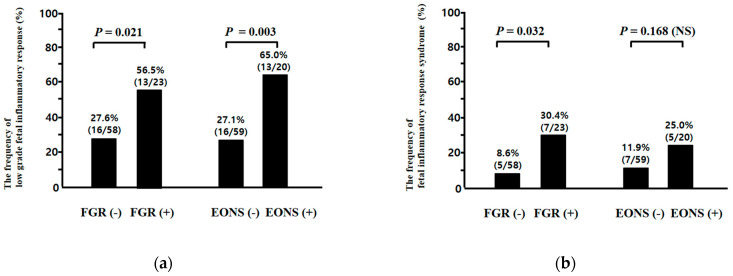
The frequency of low-grade fetal inflammatory response (FIR) (**a**) and fetal inflammatory response syndrome (FIRS) (**b**) according to the presence or absence of fetal growth restriction (FGR) (low-grade FIR, 56.5% (13/23) vs. 27.6% (16/58), *p* = 0.021; FIRS, 30.4% (7/23) vs. 8.6% (5/58), *p* = 0.032) and according to the presence or absence of early-onset neonatal sepsis (EONS) (low-grade FIR, 65.0% (13/20) vs. 27.1% (16/59), *p* = 0.003; FIRS, 25.0% (5/20) vs. 11.9% (7/59), *p* = 0.168 [NS]). Of 81 cases which met the entry for this study, two neonates were excluded from the analysis in the evaluation of EONS because they died shortly after delivery as a result of extreme prematurity, and thus only 79 neonates could be evaluated with respect to the presence or absence of EONS.

**Figure 4:**
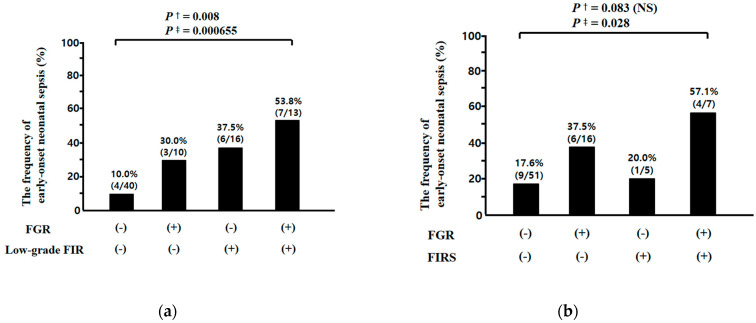
The frequency of early-onset neonatal sepsis (EONS) according to the presence or absence of fetal growth restriction (FGR) and low-grade fetal inflammatory response (FIR) (FGR[−]/low-grade FIR[−] vs. FGR[+]/low-grade FIR[−] vs. FGR[−]/low-grade FIR[+] vs. FGR[+]/low-grade FIR[+]; 10.0% (4/40) vs. 30.0% (3/10) vs. 37.5% (6/16) vs. 53.8% [7/13]) (**a**) and according to the presence or absence of FGR and fetal inflammatory response syndrome (FIRS) (FGR[−]/FIRS[−] vs. FGR[+]/FIRS[−] vs. FGR[−]/FIRS[+] vs. FGR[+]/FIRS[+]; 17.6% (9/51) vs. 37.5% (6/16) vs. 20.0% (1/5) vs. 57.1% (4/7)) (**b**). Each and every frequency or *p* value is shown in graphs; ^†^ and ^‡^ mean Pearson’s chi-square test and linear by linear association, respectively. Of 81 cases which met the entry for this study, two neonates were excluded from the analysis in the evaluation of EONS because they died shortly after delivery as a result of extreme prematurity and thus only 79 neonates could be evaluated with respect to the presence or absence of EONS.

A ROC curve was constructed to select the cut-off value at which to dichotomize the UCP CRP concentration (ng/mL) at birth to identify the subsequent development of EONS (area under curve (AUC), 0.652; standard error (SE), 0.71, *p* < 0.05), and a cut-off value of 52.8 ng/mL was selected leading to the new definition of low-grade FIR (UCP CRP concentration at birth ≥52.8 ng/mL) in the context of early preterm sterile intrauterine environment. The frequency of low-grade FIR, but not FIRS, was higher in neonates with EONS than in those without EONS (low-grade FIR, 65.0% (13/20) vs. 27.1% (16/59), *p* = 0.003; FIRS, 25.0% (5/20) vs. 11.9% (7/59), *p* = 0.168 (NS)) while both low-grade FIR and FIRS were more frequent in cases with FGR than in those without FGR (low-grade FIR, 56.5% (13/23) vs. 27.6% (16/58), *p* = 0.021; FIRS, 30.4% (7/23) vs. 8.6% (5/58), *p* = 0.032) (Figure 3a,b). There was a significantly stepwise increase in the frequency of EONS according to the presence or absence of FGR and/or low-grade FIR (FGR[−]/low-grade FIR[−] vs. FGR[+]/low-grade FIR[−] vs. FGR[−]/low-grade FIR[+] vs. FGR[+]/low-grade FIR[+]; 10.0% (4/40) vs. 30.0% (3/10) vs. 37.5% (6/16) vs. 53.8% (7/13); Pearson’s chi-square test, *p* = 0.008 and linear by linear association, *p* = 0.000655) (Figure 4a), but not FGR and/or FIRS (FGR[−]/FIRS[−] vs. FGR[+]/FIRS[−] vs. FGR[−]/FIRS[+] vs. FGR[+]/FIRS[+]; 17.6% (9/51) vs. 37.5% (6/16) vs. 20.0% (1/5) vs. 57.1% (4/7); Pearson’s chi-square test, *p* = 0.083 (NS) and linear by linear association, *p* = 0.028) (Figure 4b).

### 3.4. Clinical and Pregnant Information According to the Presence or Absence of Low-Grade Fetal Inflammatory Response (FIR) and Fetal Inflammatory Response Syndrome (FIRS)

In Supplementary Tables S1 and S2, we describe clinical and pregnancy information according to the presence or absence of low-grade FIR and FIRS, respectively. GA at delivery was significantly lower in preterm neonates with low-grade FIR than in those without low-grade FIR (Table S1), while GA at delivery was not significantly different between preterm neonates with FIRS and those without FIRS (Table S2). It should be noted that FGR was more common in neonates with low-grade FIR than in those without low-grade FIR (Table S1) and in neonates with FIRS than in those without FIRS (Table S2). However, low-grade FIR, but not FIRS, was associated with a more frequent development of EONS (Tables S1 and S2) in the context of early preterm sterile intrauterine environment.

### 3.5. Relationships between Fetal Growth Restriction (FGR) and Fetal Inflammatory Response (FIR) (i.e., Low Grade FIR and Fetal Inflammatory Response Syndrome (FIRS)), and between FIR and Early-Onset Neonatal Sepsis (EONS) in Preterm Sterile Intrauterine Environment

**Figure 5:**
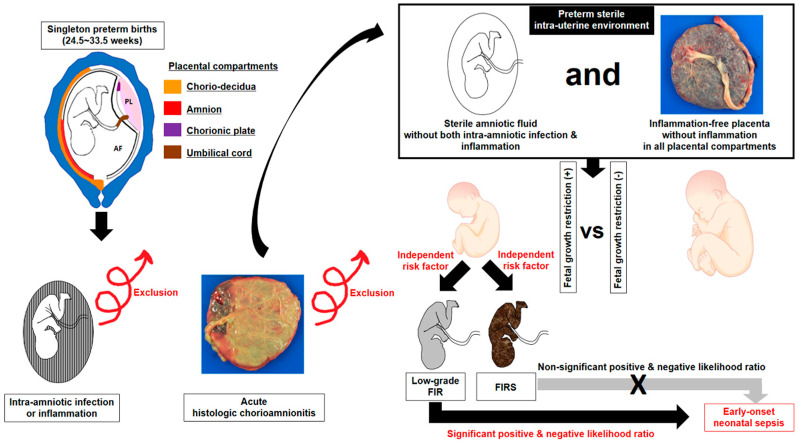
Schematic of relationships among fetal growth restriction (FGR), fetal inflammatory response (FIR) (i.e., low-grade FIR and fetal inflammatory response syndrome (FIRS)), and subsequent early-onset neonatal sepsis (EONS) in the context of early preterm sterile intrauterine environment. FGR is an independent risk factor for an increased FIR including both low-grade FIR and FIRS, and low-grade FIR, but not FIRS, had a value for the identification of EONS in the context of early preterm sterile intrauterine environment. This conceptual model is based on our current study’s data.

We performed multiple logistic regression analysis for the identification of contributing variables for the occurrence of low-grade FIR and FIRS (Table 5 and Table 6). FGR was an independent risk factor for both low-grade FIR (odds ratio (OR) 3.003, 95% confidence interval (CI) 1.024–8.812, *p* = 0.045) and FIRS (OR 4.184, 95% CI 1.101–15.902, *p* = 0.036) after the adjustment for potential confounding variables. Table 7 describes the diagnostic indices, PVs and LRs of low-grade FIR and FIRS for the identification of EONS in preterm sterile intrauterine environment. Of note, low-grade FIR had higher positive and negative PVs for the identification of EONS than FIRS did (positive PV, 44.8% (13/29) vs. 41.7% (5/12); negative PV, 86.0% (43/50) vs. 77.6% (52/67)), and moreover, low-grade FIR (positive LR and 95% CI, 2.3969 (1.4141–4.0625); negative LR and 95% CI, 0.4802 (0.2591–0.8902)), but not FIRS (positive LR and 95% CI, 2.1071 (0.7526–5.8993); negative LR and 95% CI, 0.8510 (0.6497–1.1145)), had a value for the identification of EONS (Table 7). Our results suggest a model for the relationships among FGR, FIR (i.e., low-grade FIR and FIRS), and subsequent EONS in the context of early preterm sterile intrauterine environment (Figure 5).

### 3.6. Relationship between Umbilical Cord Plasma (UCP) CRP Concentrations at Birth and Birth Weight (BW) Percentile for Gestational Age (GA) at Delivery

We examined UCP CRP concentrations at birth and BW percentile for GA at delivery. UCP CRP concentrations at birth were inversely correlated to BW percentile for GA at delivery (Spearman’s rank correlation test, *p* = 0.000698, γ = −0.369) (Figure S1).

## 4. Discussion

### 4.1. Principal Findings

Principal findings of this study were that FGR is an independent risk factor for low-grade FIR, and low-grade FIR, but not FIRS, has a value for the identification of EONS in the context of early-preterm sterile intrauterine-environment.

### 4.2. The Pathophysiology of Fetal Inflammatory Response (FIR) Associated with Fetal Growth Restriction (FGR) in the Context of Preterm Sterile Intrauterine Environment

What causes a FIR associated with FGR in the context of preterm sterile intrauterine environment? Possible explanations for this finding are as follows. Firstly, FGR is often associated with impaired blood flow to the fetus, resulting in intrauterine hypoxia [47], which can induce free radical generation at feto–maternal interface and fetal oxidative stress in in vitro studies [48,49,50,51,52]. Oxidative stress is well-known as being closely associated with the development of inflammation [53]. Secondly, in vivo animal experiments reported that the fetal liver and intestine of FGR model induced by hypoxia had the up-regulation of heat shock protein (HSP) 70 [9,12], which is known as one of prototypical damage-associated molecular patterns (DAMPs) [54]. These DAMPs are likely to be derived from stressed, injured and necrotic chorionic villi in the condition of intrauterine hypoxia associated with FGR. It is well-known that DAMPs (i.e., HSPs and S100 calcium-binding proteins) can stimulate the production of pro-inflammatory cytokines [54]. Thirdly, active maternal immune tolerance mechanisms are necessary for the tolerogenic state of paternal antigens and the prevention of rejecting fetus, because the fetus is a semi-allograft [55,56,57,58]. Therefore, detrimental recognition of the conceptus by the maternal immune system is likely to develop in some pregnancies, and this misdirected immune and inflammatory response to fetus may be associated with FGR later in pregnancy [59].

### 4.3. Relationship between Chronic Hypoxemia Absent Bacterial Infection and Fetal Inflammatory Response Syndrome (FIRS) in Animal Model

The results and preterm sterile intrauterine environment in current human study are consistent with the previous animal study [6] reporting that chronic hypoxemia absent bacterial infection is one cause of the FIRS in the following aspects. Firstly, our results display a more elevated median UCP CRP concentration in cases with FGR than in those without FGR (Figure 2a). However, a median UCP CRP concentration was much less elevated in cases with FGR in the context of preterm sterile intrauterine environment of current study than in cases with diseases (i.e., intraamniotic infection, funisitis or EONS) of another previous study [21], where about half of the study population had acute-HCA. Dong et al. also demonstrated that fetal serum IL-6 and TNF-α concentrations were elevated to only mild to moderate levels by chronic hypoxia, resulting in FGR, but those levels were robustly increased by lipopolysaccharide (LPS) stimulation in the guinea pig model [6]. These findings suggest that an increase in the FIR of preterm gestation not associated with infection is far from those levels evident during acute inflammation resulting from AIUI. Secondly, previous animal model with the use of hypoxic stimuli in the setting of sterile environment was very similar to our study population excluding both AF infection/inflammation and acute-HCA/funisitis, in that inflammatory responses were not increased in either AF or amnion [6]. It is plausible that the intensity of FIR is less potent for the subsequent penetration into the adjacent tissues and cavity (i.e., amnion and amniotic cavity) in sterile inflammation than in microbially induced inflammation. However, it should be noted that transient hypoxia in the fetal sheep stimulates transcriptomics responses that mirror inflammation, and this response may be accompanied by the appearance of bacteria in the fetal compartment of placenta, likely resulting from a hypoxia-stimulated release of bacteria from the maternal compartment to the fetal compartment of placenta due to the increased leakiness of placenta [60]. Therefore, we should recognize that an understanding of the mechanism of the physiological, cellular, and molecular responses to hypoxia may require an appreciation of stimuli other than cellular oxygen deprivation in spite of quite a small stimulus (i.e., very small numbers of bacteria not detected by culture method of AF and placenta) [60].

### 4.4. Low-Grade Fetal Inflammatory Response (FIR) and Fetal Growth Restriction (FGR) in the Context of Preterm Sterile Intrauterine Environment

Why were inflammatory responses elevated to only mild to moderate levels in cases with FGR in preterm gestation not associated with infection (Figure 2a)? One may expect that FGR is likely to share the common pathophysiology with aging-related disorders, considering “the Barker hypothesis” that FGR is associated with increased frequencies of aging-related diseases (i.e., metabolic syndrome, type 2 diabetes, atherosclerosis, and coronary heart diseases) in adult life [61]. Indeed, Davy P et al. reported that FGR was associated with accelerated telomere shortening and increased expression of cell senescence markers in the placenta [62]. Of note, aging-related disorders have the low-grade systemic inflammation in the absence overt infection (“sterile” inflammation) [63,64,65], as is the case for FGR in the current study.

### 4.5. Low-Grade Fetal Inflammatory Response (FIR) for the Identification of Early Onset Neonatal Sepsis (EONS) in the Context of Preterm Sterile Intrauterine Environment

The cut-off value (UCP CRP level at the time of delivery: 200 ng/mL) of FIRS was originally chosen to optimally identify EONS in preterm neonates, nearly half of which were born to mothers with acute-HCA in previous study [21]. However, we demonstrated that low-grade FIR had higher positive and negative PVs for the diagnosis of EONS than FIRS did, and moreover, low-grade FIR, but not FIRS, had a discriminatory value for the identification of EONS in preterm sterile intrauterine environment (Table 7). These findings suggest that the cut-off value of FIR for the identification of EONS may need to be revised lower in sterile inflammation than in microbially induced inflammation. Moreover, our results show a significantly stepwise increase in the frequency of EONS according to the presence or absence of FGR and/or low-grade FIR, but not FGR and/or FIRS (Figure 4). These results are consistent with those of previous studies reporting that FGR is a significant risk factor for EONS [66,67,68].

### 4.6. The Value for the Diagnosis of Intra-Amniotic Infection and Amniotic Fluid (AF) Inflammation by Amniocentesis to Diagnose Either Fetal Infection/Fetal Inflammatory Response Syndrome (FIRS)

In our study, for the condition of a sterile intrauterine environment, we excluded patients with at least either inflammatory AF or inflammatory placental condition as in the following: (1) positive AF culture; (2) AF inflammation; or (3) inflammation in at least one placental compartment among chorio-decidua, amnion, chorionic plate and umbilical cord. The reasons for the exclusion of either positive AF culture or AF inflammation are as follows. Firstly, positive AF culture was significantly more frequent in cases with FIRS (UCP CRP at birth >200 ng/mL) than in those without FIRS (47% vs. 20%, *p* < 0.005) [21]. Secondly, positive AF culture was significantly more frequent in cases with funisitis, the histologic counterpart of FIRS, than in those without funisitis (53% vs. 12%, *p* < 0.0001) [69]. Thirdly, patients with funisitis had a significantly higher median AF MMP-8 than those without funisitis (433.7 ng/mL vs. 1.9 ng/mL; *p* < 0.001), and the diagnostic indices of MMP-8 (cutoff, 23 ng/mL) in the identification of funisitis were sensitivity of 90%, specificity of 78%, positive predictive value of 55%, and negative predictive value of 96% [45].

### 4.7. Major Strengths and Weakness of This Study

The major strengths of this study include: (1) we excluded patients with either AF infection/inflammation or acute-HCA/funisitis, which is related to FIR [21,24,25]. Therefore, FIR in current study was thought to represent for fetal sterile inflammation not associated with AIUI; (2) it examined the relationship between low-grade FIR and FGR after controlling for potential confounding factors (i.e., GA at delivery, antenatal corticosteroids and antibiotics use, and delivery mode); and (3) we only included 81 patients through the meticulous inclusion criteria (i.e., (1) GA at delivery between 24.5 and 33.4 weeks; (2) no major congenital anomalies; (3) amniocentesis-to-delivery interval <72 h; (4) sterile AF; (5) inflammation-free placenta; and (6) available UCP CRP results at birth) among initial 2256 singleton preterm births (Figure 1). The weakness of this study is that we did not measure pathogen-associated molecular pattern’s (PAMP) antigens of microbial origin in AF. However, we additionally included inflammation-free placenta for the definition of sterile intra-uterine environment in addition to sterile AF (AF with both negative culture and negative inflammation), leading us to raise the possibility of a sterile intra-uterine environment.

### 4.8. Clinical Implication

AIUI is known to sequentially cause chorio-deciduitis, amnionitis or chorionic plate inflammation, and intraamniotic infection/inflammation, finally invading fetus (funisitis) [70,71,72]. Funisitis is thought to be the final stage of AIUI and the histologic hallmarks of FIRS [73]. Therefore, FIR was thought to be mainly associated with AIUI. However, there is cumulative evidence from in vivo preterm animal studies that FGR resulting from various stimuli (i.e., hypoxia or uterine artery ligation) in the setting absent microbial infection is associated with the increased inflammatory responses in fetal blood and organs (see Table 1) [6,7,8,9,10,11,12,13]. Therefore, fetal exposure to non-infection-related “danger-signals” inducing FGR is likely to be the one process evoking FIR. However, none of the previous human studies on the relationship between FGR and FIR were performed in a sterile intrauterine environment, and moreover, these studies had inconsistent results (see Table 2) [14,15,16,17,18,19]. Of note, our study firstly demonstrated FGR was an independent risk factor for low-grade FIR in human preterm gestation with sterile intrauterine environment, and this low-grade FIR was of value in the prediction of EONS. Our findings demonstrate that non-infection-related “danger-signals” such as FGR may play a pivotal role in the development of a sterile and low-grade FIR, and moreover, the intensity of FIR for the prediction of EONS should be revised lower in the setting of preterm sterile intrauterine environment as compared with AIUI (Figure 5).

### 4.9. Further Study

Further human research should determine if FGR is associated with the gene expression of DAMPs in inflammation-free placentas with sterile AF, and which tissues more intensely express DAMP-related genes between the feto–maternal interface or umbilical vessel wall in FGR.

## 5. Conclusions

FGR is an independent risk factor for low-grade FIR, and low-grade FIR, but not FIRS, has a value for the identification of EONS in the context of an early-preterm sterile intrauterine environment.

## Figures and Tables

**Table 1:** Relationships between the inflammatory markers of fetal blood/fetal organs and fetal growth restriction (FGR) in previous animal experiments.

Primary Author (Year), [Reference Number]	Type of Animal	n	GA at Delivery (Weeks)	GA at Stimulation of Inducing FGR (Weeks)	Stimuli ofInducing FGR	Inflammatory Markers	Intensity of Inflammatory Markers: FGR vs. Control
Fetal blood							
Dong Y (2009) [6]	Guinea pig	24	Near term	71% of term	Hypoxia	IL-6	FGR > Control
						TNF-alpha	FGR > Control
Tapanainen PJ (1994) [8]	Rat	12	Term	62% of term	Hypoxia	IGFBP-1	FGR > Control
						IGFBP-2	FGR > Control
Fetal organs							
Dong Y (2009) [6]	Guinea pig	24	Near term	71% of term	Hypoxia	* Lung	
						IL-6 (mRNA)	FGR > Control
						* Heart	
						IL-6 (mRNA)	FGR > Control
						* Brain	
						IL-6 (mRNA)	FGR > Control
						* Liver	
						IL-6 (mRNA)	FGR = Control
Guo R (2010) [7]	Guinea pig	12	Near term	71% of term	Hypoxia	* Brain (cDNA)	
						TNF-alpha	FGR > Control
						IL-1beta	FGR > Control
Zhong X (2010) [9]	Piglet	10	Term	N/A	N/A	* Intestine	
						Hsp70 (protein)	FGR > Control
						Hsp70 (mRNA)	FGR > Control
Dong Y (2011) [10]	Guinea pig	18	Near term	79% of term	Hypoxia	* Brain (mRNA)	
						iNOS	FGR > Control
Evans LC (2012) [11]	Guinea pig	132	Near term	79% of term	Hypoxia	* Heart	
						MDA (protein)	FGR > Control
						3-NT (protein)	FGR > Control
						MMP-9 (protein)	FGR > Control
Li W (2012) [12]	Piglet	10	Term	N/A	N/A	* Liver	
						T-SOD (protein)	FGR < Control
						GPx (Protein)	FGR < Control
						CAT (Protein)	FGR < Control
						MDA (protein)	FGR > Control
						Hsp70 (protein)	FGR > Control
Figueroa H (2016) [13]	Rabbit	20	Term	81% of term	Uterine artery ligation	* Kidney (mRNA)	
						iNOS	FGR > Control
						HO-1	FGR < Control
						ROS	FGR > Control
						Nitrotyrosine	FGR > Control

FGR, fetal growth restriction; GA, gestational age; N/A, not available; * means each fetal organ.

**Table 2:** Relationships between the inflammatory markers of fetal blood and fetal growth restriction (FGR) in previous human studies.

Primary Author (Year), [Reference Number]	n	GA at Delivery (Weeks)	Exclusion of Acute-HCA and AF Infection/Inflammation	Inflammatory Markers	Intensity of Inflammatory Markers: FGR vs. Control
Fetal blood					
Schiff E (1994) [14]	85	FGR: N/A Control: N/A	N/A	TNF-alpha	FGR < Control
Street ME (2006) [15]	36	FGR: 35.3 Control: 36.6	N/A	IL-6	FGR = Control
Trevisanuto D (2007) [16]	104	FGR: 34.6 Control: 34.2	N/A	hs-CRP	FGR > Control
Boutsikou T (2014) [17]	40	FGR: 37.9 Control: 38.9	N/A	hs-CRP	FGR = Control
				PAI-1	FGR = Control
				S100B	FGR = Control
Amarilyo G (2011) [18]	40	FGR: 38.2 Control: 39.1	N/A	IL-6	FGR > Control
				TNF-alpha	FGR = Control
				TPO	FGR > Control
				CRP	FGR > Control
Visentin S (2014) [19]	140	FGR: 36.75 Control: 38.57	N/A	IL-6	FGR > Control
				TNF-alpha	FGR > Control
				CRP	FGR = Control

AF, amniotic fluid; acute-HCA, acute histologic chorioamnionitis; FGR, fetal growth restriction; GA, gestational age; N/A, not available.

**Table 3:** Clinical and pregnant information of study population according to the presence or absence of fetal growth restriction (FGR).

	FGR (−) (*n* = 58) 71.6% (58/81)	FGR (+) (*n* = 23) 28.4% (23/81)	*p* Value
Mean maternal age, y (±SD)	32.1 ± 4.8	30.5 ± 4.0	0.227
Parity ≥1	56.9% (33/58)	30.4% (7/23)	0.048
Median GA at amniocentesis, week (range)	31.1 (25.9–33.4)	30.6 (25.3–33.4)	0.529
Antenatal corticosteroids use	63.8% (37/58)	73.9% (17/23)	0.443
Antibiotics use	20.7% (12/58)	17.4% (4/23)	1.000
Cesarean delivery	89.7% (52/58)	100% (23/23)	0.176
Male newborn	60.3% (35/58)	43.5% (10/23)	0.217
Causes of preterm delivery			0.024
PTL	25.9%% (15/58)	0% (0/23)	
Preterm-PROM	5.2% (3/58)	0% (0/23)	
Preeclampsia	56.9% (33/58)	87.0% (20/23)	
Other maternal fetal indication	12.1% (7/58)	13.0% (3/23)	
Median GA at delivery, week (range)	31.1 (25.9–33.4)	30.6 (25.3–33.4)	0.457
Birth weight, g (±SD)	1389 ± 395	860 ± 284	0.000
1-min Apgar score of <7	67.2% (39/58)	87.0% (20/23)	0.098
5-min Apgar score of <7	41.4% (24/58)	65.2% (15/23)	0.083
Umbilical arterial pH at birth ≤7.15 ^a^	15.8% (9/57)	27.8% (5/18)	0.303

AF, amniotic fluid; FGR, fetal growth restriction; GA, gestational age; preterm-PROM, preterm premature rupture of membranes; PTL, preterm labor and intact membranes; SD, standard deviation. ^a^ Of 81 cases which were included in the analysis of this table, seventy-five patients had an umbilical cord ABGA at birth, but 6 patients did not have an umbilical cord ABGA at birth because of the limited amount of umbilical cord arterial blood.

**Table 4:** Clinical and pregnant information of study population ^a^ according to the presence or absence of early onset neonatal sepsis (EONS).

	EONS (−) (*n* = 59) 74.7% (59/79)	EONS (+) (*n* = 20) 25.3% (20/79)	*p* Value
Mean maternal age, y (±SD)	31.9 ± 4.6	30.9 ± 4.7	0.415
Parity ≥1	54.2% (32/59)	30.0% (6/20)	0.074
Median GA at amniocentesis, week (range)	31.4 (26.9–33.4)	29.8 (25.3–32.7)	0.002
Antenatal corticosteroids use	69.5% (41/59)	60.0% (12/20)	0.583
Antibiotics use	22.0% (13/59)	15.0% (3/20)	0.749
Cesarean delivery	89.8% (53/59)	100% (20/20)	0.329
Male newborn	54.2% (32/59)	55.0% (11/20)	1.000
Causes of preterm delivery			0.410
PTL	20.3% (12/59)	10.0% (2/20)	
Preterm-PROM	5.1% (3/59)	0% (0/20)	
Preeclampsia	61.0% (36/59)	80.0% (16/20)	
Other maternal fetal indication	13.6% (8/59)	10.0% (2/20)	
Median GA at delivery, week (range)	31.4 (26.9–33.4)	29.8 (25.3–33.4)	0.002
Birth weight, g (±SD)	1356 ± 394	909 ± 408	0.000
1-min Apgar score of <7	64.4% (38/59)	95.0% (19/20)	0.009
5-min Apgar score of <7	37.3% (22/59)	75.0% (15/20)	0.004
Umbilical arterial pH at birth ≤7.15 ^b^	12.7% (7/55)	33.3% (6/18)	0.073
FGR	22.0% (13/59)	50.0% (10/20)	0.024

AF, amniotic fluid; EONS, early onset neonatal sepsis; FGR, fetal growth restriction; GA, gestational age; preterm-PROM, preterm premature rupture of membranes; PTL, preterm labor and intact membranes; SD, standard deviation. ^a^ Of 81 cases which met the entry for this study, seventy-nine patients were included in this analysis, because two neonates died shortly after delivery as a result of extreme prematurity and thus could not be evaluated with respect to the presence or absence of EONS. ^b^ Of 79 cases which were included in the analysis of this table, seventy-three patients had an umbilical cord ABGA at birth, but 6 patients did not have an umbilical cord ABGA at birth because of the limited amount of umbilical cord arterial blood.

**Table 5:** The relationship between various variables and the development of low-grade fetal inflammatory response (FIR) by multiple logistic regression analysis.

	Odds Ratio	95% CI	*p* Value
FGR	3.003	1.024–8.812	0.045
GA at delivery	0.746	0.572–0.972	0.030
Antenatal corticosteroids use	0.747	0.248–2.248	0.603
Antibiotics use	0.294	0.064–1.345	0.115
Vaginal delivery	0.000	0.000–	0.999

CI, confidence interval; FGR, fetal growth restriction; FIR, fetal inflammatory response; GA, gestational age.

**Table 6:** The relationship between various variables and the development of fetal inflammatory response syndrome (FIRS) by multiple logistic regression analysis.

	Odds Ratio	95% CI	*p* Value
FGR	4.184	1.101–15.902	0.036
GA at delivery	0.877	0.625–1.232	0.449
Antenatal corticosteroids use	1.718	0.387–7.638	0.477
Antibiotics use	0.000	0.000–	0.998
Vaginal delivery	0.000	0.000–	0.999

CI, confidence interval; FGR, fetal growth restriction; FIRS, fetal inflammatory response syndrome; GA, gestational age.

**Table 7:** Diagnostic indices, predictive values, and likelihood ratios of low-grade fetal inflammatory response (FIR; defined as an umbilical cord plasma [UCP] CRP concentration at birth ≥52.8 ng/mL in the context of early preterm sterile intrauterine milieu) and fetal inflammatory response syndrome (FIRS; defined as an UCP CRP concentration at birth ≥200 ng/mL) for the identification of proven or suspected early-onset neonatal sepsis (EONS) among preterm-neonates born to mothers with both sterile amniotic fluid (AF) and inflammation-free placenta (prevalence of proven or suspected EONS is 25.3% [20/79 ^a^]).

	Sensitivity	Specificity	Positive PV	Negative PV	Positive LR (95% CI)	Negative LR (95% CI)
Low-grade FIR	65.0% (13/20)	72.9% (43/59)	44.8% (13/29)	86.0% (43/50)	2.3969 [1.4141–4.0625]	0.4802 [0.2591–0.8902]
FIRS	25.0% (5/20)	88.1% (52/59)	41.7% (5/12)	77.6% (52/67)	2.1071 [0.7526–5.8993]	0.8510 [0.6497–1.1145]

AF, amniotic fluid; CI, confidence interval; EONS, early-onset neonatal sepsis; FIR, fetal inflammatory response; FIRS, fetal inflammatory response syndrome; LR, likelihood ratio; PV, predictive value; UCP, umbilical cord plasma. ^a^ Of 81 cases which met the entry for this study, two neonates were excluded from the analysis in the evaluation of EONS because they died shortly after delivery as a result of extreme prematurity, and thus only 79 neonates could be evaluated with respect to the presence or absence of EONS.

## Supplementary Materials

The following are available online at https://www.mdpi.com/article/10.3390/jcm10092018/s1, Table S1: Clinical and pregnant information of study population according to the presence or absence of low-grade fetal inflammatory response (FIR), Table S2: Clinical and pregnant information of study population according to the presence or absence of fetal inflammatory response syndrome (FIRS), Figure S1: Relationship between umbilical cord plasma (UCP) CRP concentrations at birth and birth weight (BW) percentile for gestational age (GA) at delivery.

## References

[1] Bamfo J.E., Odibo A.O. (2011). Diagnosis and management of fetal growth restriction. J. Pregnancy.

[2] Cunningham G.F., Leveno K.J., Bloom S.L., Spong C.Y., Dashe J.S., Hoffman B.L., Casey B.M., Sheffield J.S., Cunningham G.F., Leveno K.J., Bloom S.L., Spong C.Y., Dashe J.S., Hoffman B.L., Casey B.M., Sheffield J.S. (2014). Fetal-growth disorders. Williams Obstetrics.

[3] Hutter D., Kingdom J., Jaeggi E. (2010). Causes and mechanisms of intrauterine hypoxia and its impact on the fetal cardiovascular system: A review. Int. J. Pediatr..

[4] Krishna U., Bhalerao S. (2011). Placental insufficiency and fetal growth restriction. J. Obstet. Gynaecol. India.

[5] Baschat A.A. (2004). Fetal responses to placental insufficiency: An update. BJOG.

[6] Dong Y., Hou W., Wei J., Weiner C.P. (2009). Chronic hypoxemia absent bacterial infection is one cause of the fetal inflammatory response syndrome (FIRS). Reprod. Sci..

[7] Guo R., Hou W., Dong Y., Yu Z., Stites J., Weiner C.P. (2010). Brain injury caused by chronic fetal hypoxemia is mediated by inflammatory cascade activation. Reprod. Sci..

[8] Tapanainen P.J., Bang P., Wilson K., Unterman T.G., Vreman H.J., Rosenfeld R.G. (1994). Maternal hypoxia as a model for intrauterine growth retardation: Effects on insulin-like growth factors and their binding proteins. Pediatr. Res..

[9] Zhong X., Wang T., Zhang X., Li W. (2010). Heat shock protein 70 is upregulated in the intestine of intrauterine growth retardation piglets. Cell Stress Chaperones.

[10] Dong Y., Yu Z., Sun Y., Zhou H., Stites J., Katherine N., Weiner C.P. (2011). Chronic fetal hypoxia produces selective brain injury associated with altered nitric oxide synthases. Am. J. Obstet. Gynecol..

[11] Evans L.C., Liu H., Pinkas G.A., Thompson L.P. (2012). Chronic hypoxia increases peroxynitrite, MMP9 expression, and collagen accumulation in fetal guinea pig hearts. Pediatr. Res..

[12] Li W., Zhong X., Zhang L., Wang Y., Wang T. (2012). Heat shock protein 70 expression is increased in the liver of neonatal intrauterine growth retardation piglets. Asian Australas. J. Anim. Sci..

[13] Figueroa H., Cifuentes J., Lozano M., Alvarado C., Cabezas C., Eixarch E., Fernández E., Contreras L., Illanes S.E., Hernández-Andrade E. (2016). Nitric oxide synthase and changes in oxidative stress levels in embryonic kidney observed in a rabbit model of intrauterine growth restriction. Prenat. Diagn..

[14] Schiff E., Friedman S.A., Baumann P., Sibai B.M., Romero R. (1994). Tumor necrosis factor-alpha in pregnancies associated with preeclampsia or small-for-gestational-age newborns. Am. J. Obstet. Gynecol..

[15] Street M.E., Seghini P., Fieni S., Ziveri M.A., Volta C., Martorana D., Viani I., Gramellini D., Bernasconi S. (2006). Changes in interleukin-6 and IGF system and their relationships in placenta and cord blood in newborns with fetal growth restriction compared with controls. Eur. J. Endocrinol..

[16] Trevisanuto D., Doglioni N., Altinier S., Zaninotto M., Plebani M., Zanardo V. (2007). High-sensitivity C-reactive protein in umbilical cord of small-for-gestational-age neonates. Neonatology.

[17] Boutsikou T., Mastorakos G., Kyriakakou M., Margeli A., Hassiakos D., Papassotiriou I., Kanaka-Gantenbein C., Malamitsi-Puchner A. (2010). Circulating levels of inflammatory markers in intrauterine growth restriction. Mediat. Inflamm..

[18] Amarilyo G., Oren A., Mimouni F.B., Ochshorn Y., Deutsch V., Mandel D. (2011). Increased cord serum inflammatory markers in small-for-gestational-age neonates. J. Perinatol..

[19] Visentin S., Lapolla A., Londero A.P., Cosma C., Dalfrà M., Camerin M., Faggian D., Plebani M., Cosmi E. (2014). Adiponectin levels are reduced while markers of systemic inflammation and aortic remodelling are increased in intrauterine growth restricted mother-child couple. BioMed Res. Int..

[20] Gomez R., Romero R., Ghezzi F., Yoon B.H., Mazor M., Berry S.M. (1998). The fetal inflammatory response syndrome. Am. J. Obstet. Gynecol..

[21] Yoon B.H., Romero R., Shim J.Y., Shim S.S., Kim C.J., Jun J.K. (2003). C-reactive protein in umbilical cord blood: A simple and widely available clinical method to assess the risk of amniotic fluid infection and funisitis. J. Matern. Fetal Neonatal Med..

[22] Gotsch F., Romero R., Kusanovic J.P., Mazaki-Tovi S., Pineles B.L., Erez O., Espinoza J., Hassan S.S. (2007). The fetal inflammatory response syndrome. Clin. Obstet. Gynecol..

[23] Savasan Z.A., Chaiworapongsa T., Romero R., Hussein Y., Kusanovic J.P., Xu Y., Dong Z., Kim C.J., Hassan S.S. (2012). Interleukin-19 in fetal systemic inflammation. J. Matern. Fetal Neonatal Med..

[24] Lee S.E., Romero R., Jung H., Park C.W., Park J.S., Yoon B.H. (2007). The intensity of the fetal inflammatory response in intraamniotic inflammation with and without microbial invasion of the amniotic cavity. Am. J. Obstet. Gynecol..

[25] Park C.W., Moon K.C., Park J.S., Jun J.K., Romero R., Yoon B.H. (2009). The involvement of human amnion in histologic chorioamnionitis is an indicator that a fetal and an intra-amniotic inflammatory response is more likely and severe: Clinical implications. Placenta.

[26] Park C.W., Yoon B.H., Park J.S., Jun J.K. (2013). A fetal and an intra-amniotic inflammatory response is more severe in preterm labor than in preterm prom in the context of funisitis: Unexpected observation in human gestations. PLoS ONE.

[27] Park C.W., Yoon B.H., Park J.S., Jun J.K. (2013). An elevated maternal serum C-reactive protein in the context of intra-amniotic inflammation is an indicator that the development of amnionitis, an intense fetal and af inflammatory response are likely in patients with preterm labor: Clinical implications. J. Matern. Fetal Neonatal Med..

[28] Park C.W., Kim S.M., Park J.S., Jun J.K., Yoon B.H. (2014). Fetal, amniotic and maternal inflammatory responses in early stage of ascending intrauterine infection, inflammation restricted to chorio-decidua, in preterm gestation. J. Matern. Fetal Neonatal Med..

[29] Park C.W., Park J.S., Moon K.C., Jun J.K., Yoon B.H. (2016). Preterm labor and preterm premature rupture of membranes have a different pattern in the involved compartments of acute histologoic chorioamnionitis and/or funisitis: Patho-physiologic implication related to different clinical manifestations. Pathol. Int..

[30] Oh J.W., Park C.W., Moon K.C., Park J.S., Jun J.K. (2019). The relationship among the progression of inflammation in umbilical cord, fetal inflammatory response, early-onset neonatal sepsis, and chorioamnionitis. PLoS ONE.

[31] Oh J.W., Park C.W., Moon K.C., Park J.S., Jun J.K. (2020). Fetal inflammatory response is positively correlated with the progress of inflammation in chorionic plate. Placenta.

[32] Hofer N., Kothari R., Morris N., Müller W., Resch B. (2013). The fetal inflammatory response syndrome is a risk factor for morbidity in preterm neonates. Am. J. Obstet. Gynecol..

[33] Sursal T., Stearns-Kurosawa D.J., Itagaki K., Oh S., Sun S., Kurosawa S., Hauser C.J. (2013). Plasma bacterial and mito-chondrial DNA distinguish bacterial sepsis from sterile systemic inflammatory response syndrome and quantify in-flammatory tissue injury in nonhuman primates. Shock.

[34] Kaplan L.J. (2019). Systemic Inflammatory Response Syndrome. Medscape, eMedicine. http://www.emedicine.medscape.com/article/168943-overview.

[35] Horeczko T., Green J.P., Panacek E.A. (2014). Epidemiology of the systemic inflammatory response syndrome (SIRS) in the emergency department. West. J. Emerg. Med..

[36] Yoon B.H., Yang S.H., Jun J.K., Park K.H., Kim C.J., Romero R. (1996). Maternal blood C-reactive protein, white blood cell count, and temperature in preterm labor: A comparison with amniotic fluid white blood cell count. Obstet. Gynecol..

[37] Yoon B.H., Jun J.K., Park K.H., Syn H.C., Gomez R., Romero R. (1996). Serum C-reactive protein, white blood cell count, and amniotic fluid white blood cell count in women with preterm premature rupture of membranes. Obstet. Gynecol..

[38] ACOG Committee on Practice Bulletins-Obstetrics (2004). ACOG Practice Bulletin. Clinical management guidelines for obste-tricians-gynecologists. Management of postterm pregnancy. Obstet. Gynecol..

[39] Zhang J., Mikolajczyk R., Grewal J., Neta G., Klebanoff M. (2011). Prenatal application of the individualized fetal growth reference. Am. J. Epidemiol..

[40] McIntire D.D., Bloom S.L., Casey B.M., Leveno K.J. (1999). Birth weight in relation to morbidity and mortality among new born infants. N. Engl. J. Med..

[41] Doubilet P.M., Benson C.B., Nadel A.S., Ringer S.A. (1997). Improved birth weight table for neonates developed from ges-tations dated by early ultrasonography. J. Ultrasound Med..

[42] Stoll B.J., Hansen N.I., Adams-Chapman I., Fanaroff A.A., Hintz S.R., Vohr B., Higgins R.D. (2004). National institute of child health and human development neonatal research network. neurodevelopmental and growth impairment among extremely low-birth-weight infants with neonatal infection. JAMA.

[43] Yoon B.H., Romero R., Kim C.J., Jun J.K., Gomez R., Choi J.H., Syn H.C. (1995). Amniotic fluid interleukin-6: A sensitive test for antenatal diagnosis of acute inflammatory lesions of preterm placenta and prediction of perinatal morbidity. Am. J. Obstet. Gynecol..

[44] Park C.W., Yoon B.H., Kim S.M., Park J.S., Jun J.K. (2013). The frequency and clinical significance of intra-amniotic inflam-mation defined as an elevated amniotic fluid matrix metalloproteinase-8 in patients with preterm labor and low amniotic fluid white blood cell counts. Obstet. Gynecol. Sci..

[45] Park J.S., Romero R., Yoon B.H., Moon J.B., Oh S.Y., Han S.Y., Ko E.M. (2001). The relationship between amniotic fluid matrix metalloproteinase-8 and funisitis. Am. J. Obstet. Gynecol..

[46] Shim S.S., Romero R., Hong J.S., Park C.W., Jun J.K., Kim B.I., Yoon B.H. (2004). Clinical significance of intra-amniotic in-flammation in patients with preterm premature rupture of membranes. Am. J. Obstet. Gynecol..

[47] Longini M., Perrone S., Kenanidis A., Vezzosi P., Marzocchi B., Petraglia F., Centini G., Buonocore G. (2005). Isoprostanes in amniotic fluid: A predictive marker for fetal growth restriction in pregnancy. Free Radic. Biol. Med..

[48] Kamath U., Rao G., Kamath S.U., Rai L. (2006). Maternal and fetal indicators of oxidative stress during intrauterine growth retardation (IUGR). Indian J. Clin. Biochem..

[49] Hracsko Z., Safar Z., Orvos H., Novak Z., Pal A., Varga I.S. (2007). Evaluation of oxidative stress markers after vaginal delivery or Caesarean section. In Vivo.

[50] Mert I., Oruc A.S., Yuksel S., Cakar E.S., Buyukkagnici U., Karaer A., Danisman N. (2012). Role of oxidative stress in preeclampsia and intrauterine growth restriction. J. Obstet. Gynaecol. Res..

[51] Biri A., Bozkurt N., Turp A., Kavutcu M., Himmetoglu O., Durak I. (2007). Role of oxidative stress in intrauterine growth restriction. Gynecol. Obstet. Investig..

[52] Karowicz-Bilinska A., Kedziora-Kornatowska K., Bartosz G. (2007). Indices of oxidative stress in pregnancy with fetal growth restriction. Free Radic. Res..

[53] Reuter S., Gupta S.C., Chaturvedi M.M., Aggarwal B.B. (2010). Oxidative stress, inflammation, and cancer: How are they linked?. Free Radic. Biol. Med..

[54] Chen G.Y., Nuñez G. (2010). Sterile inflammation: Sensing and reacting to damage. Nat. Rev. Immunol..

[55] Leber A., Teles A., Zenclussen A.C. (2010). Regulatory T cells and their role in pregnancy. Am. J. Reprod. Immunol..

[56] Petroff M.G., Perchellet A. (2010). B7 family molecules as regulators of the maternal immune system in pregnancy. Am. J. Reprod. Immunol..

[57] Lee J., Romero R., Chaiworapongsa T., Dong Z., Tarca A.L., Xu Y., Chiang P.J., Kusanovic J.P., Hassan S.S., Yeo L. (2013). Characterization of the fetal blood transcriptome and proteome in maternal anti-fetal rejection: Evidence of a distinct and novel type of human fetal systemic inflammatory response. Am. J. Reprod. Immunol..

[58] Mor G., Cardenas I. (2010). The immune system in pregnancy: A unique complexity. Am. J. Reprod. Immunol..

[59] Girardi G., Yarilin D., Thurman J.M., Holers V.M., Salmon J.E. (2006). Complement activation induces dysregulation of an-giogenic factors and causes fetal rejection and growth restriction. J. Exp. Med..

[60] Wood C.E., Keller-Wood M. (2019). Current paradigms and new perspectives on fetal hypoxia: Implications for fetal brain development in late gestation. Am. J. Physiol. Regul. Integr. Comp. Physiol..

[61] Barker D.J.P. (1998). Mothers, Babies, and Health in Later Life.

[62] Davy P., Nagata M., Bullard P., Fogelson N.S., Allsopp R. (2009). Fetal growth restriction is associated with accelerated te-lomere shortening and increased expression of cell senescence markers in the placenta. Placenta.

[63] Franceschi C., Campisi J. (2014). Chronic inflammation (inflammaging) and its potential contribution to age-associated dis-eases. J. Gerontol. A Biol. Sci. Med. Sci..

[64] Sarkar D., Fisher P.B. (2006). Molecular mechanisms of aging-associated inflammation. Cancer Lett..

[65] Franceschi C., Bonafè M., Valensin S., Olivieri F., De Luca M., Ottaviani E., De Benedictis G. (2000). Inflamm-aging. An evolutionary perspective on immunosenescence. Ann. N. Y. Acad. Sci..

[66] Damodaram M., Story L., Kulinskaya E., Rutherford M., Kumar S. (2011). Early adverse perinatal complications in preterm growth-restricted fetuses. Aust. N. Z. J. Obstet. Gynaecol..

[67] Engineer N., Kumar S. (2010). Perinatal variables and neonatal outcomes in severely growth restricted preterm fetuses. Acta Obstet. Gynecol. Scand..

[68] Simchen M.J., Beiner M.E., Strauss-Liviathan N., Dulitzky M., Kuint J., Mashiach S., Schiff E. (2000). Neonatal outcome in growth-restricted versus appropriately grown preterm infants. Am. J. Perinatol..

[69] Yoon B.H., Romero R., Park J.S., Kim M., Oh S.Y., Kim C.J., Jun J.K. (2000). The relationship among inflammatory lesions of the umbilical cord (funisitis), umbilical cord plasma interleukin 6 concentration, amniotic fluid infection, and neonatal sepsis. Am. J. Obstet. Gynecol..

[70] Goldenberg R.L., Hauth J.C., Andrews W.W. (2000). Intrauterine infection and preterm delivery. N. Engl. J. Med..

[71] Romero R., Mazor M. (1988). Infection and preterm labor. Clin. Obstet. Gynecol..

[72] Goncalves L.F., Chaiworapongsa T., Romero R. (2002). Intrauterine infection and prematurity. Ment. Retard. Dev. Disabil. Res. Rev..

[73] Pacora P., Chaiworapongsa T., Maymon E., Kim Y.M., Gomez R., Yoon B.H., Ghezzi F., Berry S.M., Qureshi F., Jacques S.M. (2002). Funisitis and chorionic vasculitis: The histological counterpart of the fetal inflammatory response syndrome. J. Matern. Fetal Neonatal Med..

