# Supplementary material for: Fetal Growth Restriction and Subsequent Low Grade Fetal Inflammatory Response Are Associated with Early-Onset Neonatal Sepsis in the Context of Early Preterm Sterile Intrauterine Environment
Source: J Clin Med. 2021 May 8;10(9):2018. doi: 10.3390/jcm10092018 (PMC8125902; doi:10.3390/jcm10092018)
Supplement: Supplementary file 1 [file jcm-10-02018-s001.zip › jcm-1166034-supplementary.pdf]

Supplementary Materials

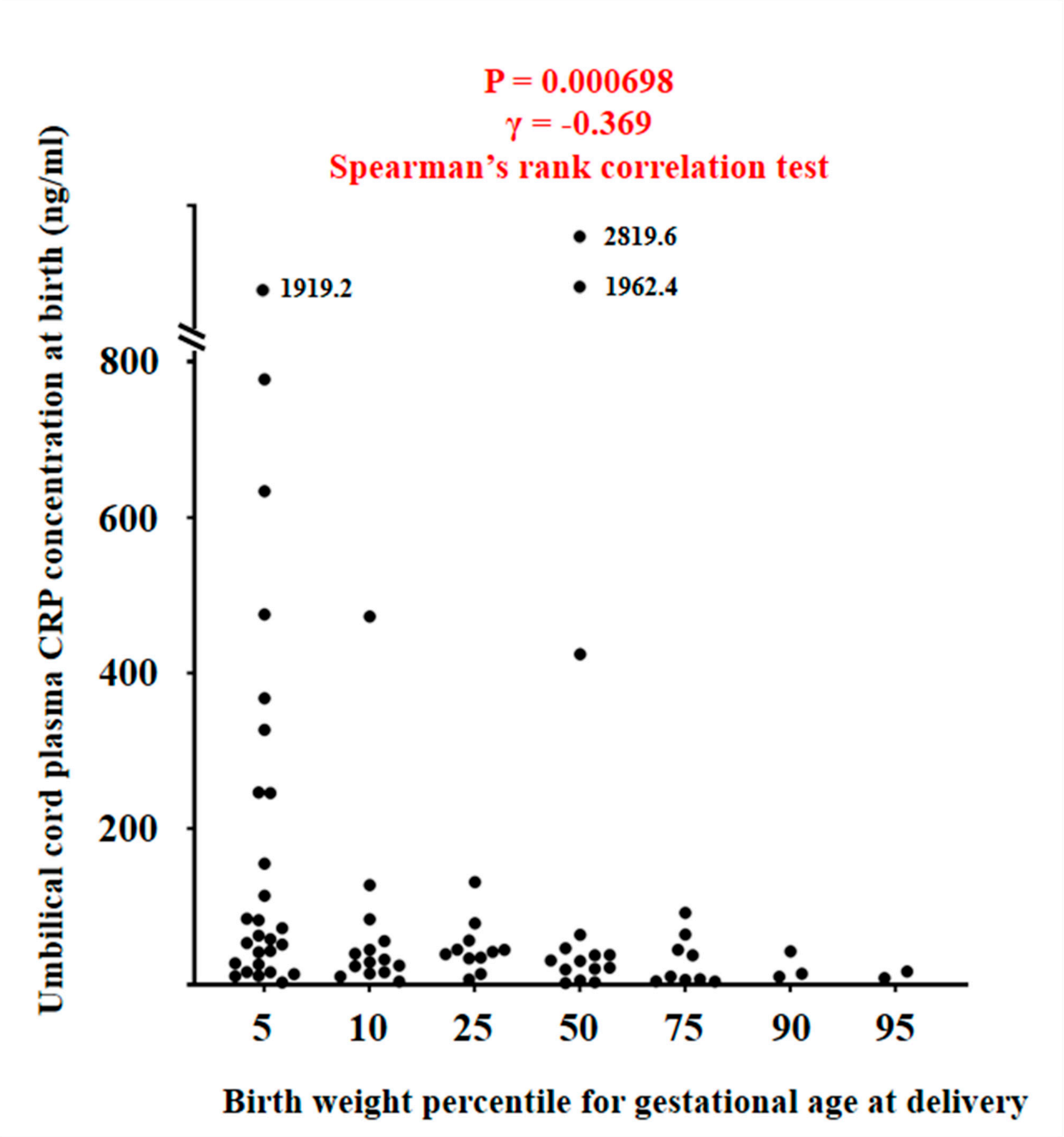

Supplementary Figure 1 (Figure S1). Relationship between umbilical cord plasma (UCP) CRP concentrations at birth and birth weight (BW) percentile for gestational age (GA) at delivery. UCP CRP concentrations at birth were inversely correlated to BW percentile for GA at delivery (Spearman's rank correlation test, P=0.000698, γ=-0.369).

Supplementary Table 1. Clinical and pregnant information of study population according to the presence or absence of low-grade fetal inflammatory response (FIR)

	Low-grade FIR (-) UCP CRP < 52.8 ng/ml (n=52) 64.2% (52/81)	Low-grade FIR (+) UCP CRP ≥ 52.8 ng/ml (n=29) 35.8% (29/81)	*P* value
Mean maternal age, y (± SD)	31.9 ± 4.4	31.2 ± 4.9	0.653
Parity ≥1	55.8% (29/52)	37.9% (11/29)	0.165
Median GA at amniocentesis, week (range)	31.3 (25.3-33.4)	30.3 (25.9-33.4)	0.035
Antenatal corticosteroids use	67.3% (35/52)	65.5% (19/29)	1.000
Antibiotics use	25.0% (13/52)	10.3% (3/29)	0.150
Cesarean delivery	88.5% (46/52)	100% (29/29)	0.083
Male newborn	61.5% (32/52)	44.8% (13/29)	0.168
Causes of preterm delivery			0.092
PTL or preterm-PROM	28.8% (15/52)	10.3% (3/29)	
Maternal fetal indication	71.2% (37/52)	89.7% (26/29)	
Median GA at delivery, week (range)	31.4 (25.3-33.4)	30.3 (25.9-33.4)	0.027
Birth weight, g (± SD)	1370 ± 440	1002 ± 321	0.000
1-min Apgar score of < 7	67.3% (35/52)	82.8% (24/29)	0.193
5-min Apgar score of < 7	40.4% (21/52)	62.1% (18/29)	0.069
Umbilical arterial pH at birth ≤ 7.15[a]	15.7% (8/51)	25.0% (6/24)	0.355
FGR	19.2% (10/52)	44.8% (13/29)	0.021
EONS[b]	14.0% (7/50)	44.8% (13/29)	0.003

AF, amniotic fluid; *CRP*, C-reactive protein; *EONS*, early-onset neonatal sepsis; *FGR*, fetal growth restriction; *FIR*, fetal inflammatory response; *GA*, gestational age; *preterm-PROM*, preterm premature rupture of membranes; *PTL*, preterm labor and intact membranes; *SD*, standard deviation; *UCP*, umbilical cord plasma

[a] Of 81 cases which were included in the analysis of this table, seventy-five patients had an umbilical cord ABGA at birth, but 6 patients did not have an umbilical cord ABGA at birth because of the limited amount of umbilical cord arterial blood.

[b] Of 81 cases which met the entry for this study, seventy-nine patients were included in this analysis, because two neonates died shortly after delivery as a result of extreme prematurity and thus could not be evaluated with respect to the presence or absence of EONS.

Supplementary Table 2. Clinical and pregnant information of study population according to the presence or absence of fetal inflammatory response syndrome (FIRS)

	FIRS (-)<br>UCP CRP < 200 ng/ml<br>(n=69)<br>85.2% (69/81)	FIRS (+)<br>UCP CRP ≥ 200 ng/ml<br>(n=12)<br>14.8% (12/81)	*P* value
Mean maternal age, y (± SD)	32.0 ± 4.4	29.8 ± 5.4	0.140
Parity ≥1	53.6% (37/69)	25.0% (3/12)	0.116
Median GA at amniocentesis, week (range)	31.1 (25.3-33.4)	30.7 (26.9-32.6)	0.406
Antenatal corticosteroids use	65.2% (45/69)	75.0% (9/12)	0.742
Antibiotics use	23.2% (16/69)	0% (0/12)	0.111
Cesarean delivery	91.3% (63/69)	100% (12/12)	0.584
Male newborn	59.4% (41/69)	33.3% (4/12)	0.121
Causes of preterm delivery			0.283
PTL or preterm-PROM	24.6% (17/69)	8.3% (1/12)	
Maternal fetal indication	75.4% (52/69)	91.7% (11/12)	
Median GA at delivery, week (range)	31.1 (25.3-33.4)	30.7 (26.9-32.6)	0.365
Birth weight, g (± SD)	1288 ± 445	956 ± 241	0.006
1-min Apgar score of < 7	72.5% (50/69)	75.0% (9/12)	1.000
5-min Apgar score of < 7	46.4% (32/69)	58.3% (7/12)	0.538
Umbilical arterial pH at birth ≤ 7.15[a]	19.4% (13/67)	12.5% (1/8)	1.000
FGR	23.2% (16/69)	58.3% (7/12)	0.032
EONS[b]	22.4% (15/67)	41.7% (5/12)	0.168

AF, amniotic fluid; *CRP*, C-reactive protein; *EONS*, early-onset neonatal sepsis; *FGR,* fetal growth restriction; *FIRS*, fetal inflammatory response syndrome; *GA*, gestational age; *preterm-PROM*, preterm premature rupture of membranes; *PTL*, preterm labor and intact membranes; *SD*, standard deviation; *UCP*, umbilical cord plasma

[a] Of 81 cases which were included in the analysis of this table, seventy-five patients had an umbilical cord ABGA at birth, but 6 patients did not have an umbilical cord ABGA at birth because of the limited amount of umbilical cord arterial blood.

[b] Of 81 cases which met the entry for this study, seventy-nine patients were included in this analysis, because two neonates died shortly after delivery as a result of extreme prematurity and thus could not be evaluated with respect to the presence or absence of EONS.
